# Strengthening the emergency healthcare system for mothers and children in The Gambia

**DOI:** 10.1186/1742-4755-7-21

**Published:** 2010-08-18

**Authors:** Ramou Cole-Ceesay, Meena Cherian, Alieu Sonko, Nestor Shivute, Mamady Cham, Michael Davis, Famara Fatty, Susan Wieteska, Momodou Baro, Diane Watson, Barbara Phillips, Rhona MacDonald, Brigid Hayden, David Southall

**Affiliations:** 1 The State Department, Government of The Gambia, Banjul, The Gambia; 2 Emergency & Essential Surgical Care Clinical Procedures Unit (CPR), Department of Essential Health Technologies, WHO Geneva, Switzerland; 3 Head Office, Brikama Major Health Centre, Brikama, The Gambia; 4 HO Country Office, PMB 170, Banjul, The Gambia; 5 The Ministry of Health, The Gambian Government, Banjul, The Gambia; 6 Education Department, The Advanced Life Support Group, 29-31 Ellesmere Street, Manchester, M27 0LA, UK; 7 Famara Fatty Reproductive and Child Health Unit, The Ministry of Health, Banjul, The Gambia; 8 Head Office, The Advanced Life Support Group, 29-31 Ellesmere Street, Manchester M27 0LA, UK; 9 Anaesthetic Department, Royal Victoria Teaching Hospital, Banjul, The Gambia; 10 Anaesthetic Department, Royal Gwent Hospital, Newport, South Wales, UK; 11 Head Office, Maternal and Childhealth Advocacy International (MCAI), Conway Chambers, 83 Derby Rd, Nottingham NG1 5BB, UK

## Abstract

A system to improve the management of emergencies during pregnancy, childbirth, infancy and childhood in a region of The Gambia (Brikama) with a population of approximately 250,000 has been developed.

This was accomplished through formal partnership between the Gambian Ministry of Health, the World Health Organisation, Maternal Childhealth Advocacy International and the Advanced Life Support Group.

Since October 2006, the hospital in Brikama has been renovated and equipped and more efficiently provided with emergency medicines. An emergency ambulance service now links the community with the hospital through a mobile telephone system. Health professionals from community to hospital have been trained in obstetric, neonatal and paediatric emergency management using skills' based education. The programme was evaluated in log books detailing individual resuscitations and by external assessment.

The hospital now has constant water and electricity, a functioning operating theatre and emergency room; the maternity unit and children's wards have better emergency equipment and there is a more reliable supply of oxygen and emergency drugs, including misoprostol (for treating post partum haemorrhage) and magnesium sulphate (for severe pre-eclampsia). There is also a blood transfusion service.

Countrywide, 217 doctors, nurses, and midwives have undergone accredited training in the provision of emergency maternal, newborn and child care, including for major trauma. 33 have received additional education through Generic Instructor Courses and 15 have reached full instructor status. 83 Traditional Birth Attendants and 48 Village Health Workers have been trained in the recognition and initial management of emergencies, including resuscitation of the newborn. Eleven and ten nurses underwent training in peri-operative nursing and anaesthetics respectively, to address the acute shortage required for emergency Caesarean section.

Between May 2007 and March 2010, 109 patients, mostly pregnant mothers, were stabilised and transported to hospital by the new emergency ambulance service.

293 resuscitation attempts were documented in personal logbooks.

A sustainable system for better managing emergencies has been established and is helping to negate the main obstacle impeding progress: the country's lack of available trained medical and nursing staff. However, insufficient attention was paid to improving staff morale and accommodation representing significant failings of the programme.

## Introduction

The Gambia has a population of 1.663 million and in 2006 around 60,000 births per year [1]. The per capita income is US$310 per annum [1]. In 2005, it had a high adjusted maternal mortality ratio of 690/100,000 live births, a 30% decline from the 1990 levels. The infant mortality rate in 2006 was 84/1000 and under 5 year mortality 113/1,000 live births [1]. Institutional delivery is estimated at 55% (2000-2006) [1]. A significant proportion of births, especially in rural areas, are conducted in villages by Traditional Birth Attendants (TBAs) with limited training and little support.

The most common life-threatening emergencies [2] in mothers and children are 1) pregnancy related: massive peri-partum haemorrhage, eclampsia and sepsis. 2) in newborn infants: failure to breathe at birth and sepsis 3) in children; malaria, pneumonia, malnutrition and diarrhoeal disease, 4) at all ages major trauma such as burns and road traffic accidents. The indirect causes of morbidity and mortality are severe anaemia and malaria. Contributing factors are multiple and include low literacy levels of women, high attrition rate and low morale of skilled health workers, and poverty.

To date, the international health community has mainly focused on interventions to promote maternal and child health through primary prevention but, inevitably, emergency situations will arise, especially in pregnancy and early childhood. In poorly resourced countries lacking basic health infrastructure in the Public National Health service (NHS), emergencies are often poorly managed leading to avoidable maternal and child deaths and great morbidity and misery [3-6].

Although there has been much work in identifying the factors contributing to deaths from emergencies, especially those occurring around childbirth itself [5,7,8], sustainable and effective solutions are difficult to achieve.

The difficulty arises from the nature of emergency care. Emergencies are largely unpredictable and any system to manage them must be available at all times. Safe and effective emergency care relies on an adequately functioning healthcare system with minimal delays at all levels. The emergency system has several components, all of which must function at least adequately if the "emergency chain of care" is to be reliable and sustainable. The initial step is the recognition that there is an emergency need. The next step is community first aid, effective communication and reliable transport to an adequately functioning and equipped health facility with appropriately trained and skilled staff, immediate effective treatment and onward clinically safe transport to a higher level facility if necessary. All components must be maintained in a state of readiness and developed by audit and feedback.

However, in poorly resourced countries the underlying infrastructure to support good communication and transport is often lacking and inadequate drug procurement and distribution deprives the health care system of medical supplies and emergency treatment. There is a major shortage of skilled staff in the public health service and motivation, societal customs and previous adverse experiences lead the patient and his/her family to delay or even avoid the decision to access health care [9].

In 2006, following a countrywide tour of healthcare facilities by WHO/Gambia and the Ministry of Health (MOH) agreement was reached to improve access to essential surgical care, especially emergency obstetric care, in publically funded Major Health Centres and Hospitals.

The Unit at the WHO headquarters in Geneva responsible for the Emergency and Essential Surgical Care project and the development of the Integrated Management of Emergency and Essential Surgical Care (IMEESC) toolkit [10], consulted with the MOH which supported a situation analysis to address gaps in surgical care (including emergency and anaesthesia services).

In April 2006, a Joint WHO and MOH Meeting was held with key policy makers and health providers. The recommendations included joint activities with Maternal and Childhealth Advocacy International (MCAI) and the Advanced Life Support Group (ALSG), two UK international medical organisations, working in partnership with the MOH and WHO Country Office, Gambia, to improve emergency and surgical care through incorporation of the Strengthening Emergency Healthcare programme developed by MCAI and ALSG [11,12]. This program had been successfully piloted in Pakistan [13].

In June 2007, a two day joint WHO and MOH workshop on the use of the WHO IMEESC toolkit was conducted for key health providers representing all major and some minor health centres facilitated by two senior health providers (Nurse Anaesthetist MB and a Consultant Surgeon) from the Royal Victoria Teaching Hospital (RVTH) in The Gambia.

Technical assistance was provided by WHO in teaching and training tools such as IMEESC toolkits containing 5 CD ROMs and WHO manuals "Surgical Care at the District Hospital" [10], advocacy materials and handouts on best practice protocols for clinical procedures, emergency equipment list, monitoring and evaluation tools[10] to all participants.

A country-wide assessment of emergency services was undertaken in July 2006 by the 4 partners (MOH, WHO, MCAI, and ALSG). This revealed that hospitals lacked adequate electrical power, regular water supplies, basic equipment, emergency drugs and medical supplies. Health workers were found to lack the skills needed to manage emergencies, particularly those related to obstetric complications, resuscitation at birth and life threatening problems in newborn infants, serious paediatric illnesses and major trauma. There was little Continuing Medical Education and a poor state of morale and motivation in health workers of all levels. Many Government trained nurses, midwives and doctors had left the health service for the private sector or to work for international organisations where they were paid more and had much better work incentives and accommodation.

In October 2006, a pilot programme began in the Brikama region, in which there was a poorly functioning Major Health Centre (District Hospital) serving a population of around 250,000 people. This was initiated by the signing of a Memorandum of Understanding (MOU).

The programme's goal was that all emergencies be effectively managed within the Gambian NHS with minimal delay, 24 hours a day, through integration between the community and a basically equipped and adequately staffed Hospital with an operating theatre supported by a functioning laboratory, including blood banking and safe transfusion.

## Discussion

The programme began in October 2006 and is continuing today-25th July 2010.

### 1. Strengthening the emergency healthcare system within Brikama hospital

To assist with cleanliness, patient dignity, safety and efficiency and to improve staff morale, renovation was undertaken by local builders employed by the MOH. Problems with the plumbing and electrical fitments were partially corrected. Many fans, light bulbs, sinks, toilets and showers that were not working were repaired. Unfortunately, many of these repairs were of poor quality and essential electrical supplies, such as light bulbs, remained intermittently available and toilets and sinks frequently became unusable. Following consultation with the local community, the hospital is now receiving support to address these problems from local fundraising activities. Extensive painting and decorating was undertaken, including work to make the children's wards more "child friendly" [14,15]

Largely successful attempts were made to ensure the continuous and sustainable provision of essential emergency drugs (including oxygen) and medical/surgical supplies.

Essential basic emergency equipment required was sourced from the UK. Some was provided second-hand from UK hospitals at auction including high quality beds, washable mattresses, an operating table, theatre trolley, resuscitation and drugs trolleys, examination couches, and suction systems. However, more complex electrical items which might require regular servicing, such as oxygen concentrators, air conditioners, an ultrasound scanner and anaesthetic machine were purchased new with service contracts from manufacturers.

There was no hospital security system. There was regular harassment of staff by relatives. Thefts and assaults on staff occurred, especially at night and animals regularly wandered into the hospital wards. The MOH with the assistance of the local Governor, established 24 hour security, assisted by a new gate and quarters for security guards.

An existing but disused operating room was renovated and is currently supported by an effective blood transfusion service and appropriately trained staff [11]. Renovations included tiling of the floor and walls, new plumbing, installation of effective lighting, 2 new air conditioners, sufficient electrical plugs protected by voltage regulator systems, and renovation of an old wall oxygen system to provide a continuous supply from 2 large cylinders. An autoclave, cautery and powerful suction systems, an anaesthetic machine (Glostavent) appropriate for use in resource constrained settings, wall clocks, head torches, chest drain kits, sets of surgical instruments, bag valve mask resuscitators, oropharyngeal airways, stethoscopes and a high powered re-chargeable torch were provided new from manufacturers in the UK. A manual hydraulic operating table, stainless steel trolleys and stools, portable theatre lights, pulse oximeter, automatic blood pressure machine and autoclave were provided (all high quality second hand). A scrub/preparation room was established including a sink with elbow operated taps. A blood bank and pharmacy refrigerator, a changing room, toilet and shower, and storage facilities for equipment and disposables were setup. A recovery area was established containing two second hand emergency beds, an incubator and resuscitation equipment, a new sink and wall oxygen supplies.

The laboratory service was upgraded to provide 24 hours a day grouping and cross-match of blood for transfusion. A second hand refrigerator to store blood and community sensitisation to encourage blood donors has resulted in a blood transfusion bank. Nevertheless, most donations for transfusions continue to be from relatives accompanying patients experiencing emergency conditions.

The labour ward and postnatal areas were both unhygienic and poorly equipped [11]. Labour ward beds, baby cots, automatic blood pressure machines, theatre and drug trolleys, beds and mattresses, pulse oximeters, neonatal resuscitaire, and suction equipment (all high quality second hand) were provided. An oxygen concentrator, bag valve mask resuscitators for babies and adults, a wall clock, surgical instruments, vacuum extractor, portable electronic fetal heart rate monitors, ultrasound scanner, oropharyngeal airways and digital and forehead thermometers were provided new from the manufacturers.

Emergency drugs (identified from the Gambia, WHO essential drug list) such as antibiotics, oxygen, adrenaline, opiate analgesics, and fluids, together with emergency medical supplies were provided by the Central Pharmacy only after orders had been placed. Systems to improve the ordering of these items so that supplies did not run-out, was only partially successful, especially with respect to oxygen cylinders which were frequently empty and not available for emergency care.

Additional essential emergency drugs such as magnesium sulphate and misoprostol have been made available for treating eclampsia and post-partum haemorrhage respectively in the maternity unit and in the emergency kit carried by the emergency ambulance service midwives (see below). The provision of these two new drugs was supported by one day training courses organised by Gambian Instructors.

Attempts were made to enhance a system that aims to provide an effective bioengineering service for medical and surgical equipment throughout the country. However, this service remains a problem, especially outside the Royal Victoria Teaching Hospital (RVTH) in Banjul.

Laminated wall charts documenting pathways of care for emergency conditions that might occur were provided and are available for download [16]. For example the labour ward carries charts on the management of massive obstetric haemorrhage, prolapsed cord, eclampsia, neonatal resuscitation, shoulder dystocia and shock.

### 2. Integration of emergency care between community villages and the hospital

Prior to this programme, all patients from the Brikama region had to be transferred to RVTH (a journey of at least 30 minutes) if they needed a Caesarean Section or other surgical intervention. This caused delays with avoidable complications and deaths.

The area to be covered by this upgraded emergency system involved a 30 mile radius around the hospital at Brikama and included some poor quality roads, especially in the rainy season.

To assist with continuity of care and minimise delays, an emergency ambulance system was designed. This was run by 2 midwives who had completed the EMNH course (see below), (Mrs J Fatty and Mr L Darboe) providing cover 24 hours a day, 7 days a week. They each carried an emergency kit [17] and were transported in a dedicated ambulance (donated by The President of The Gambia) with driver (appointed by the "Riders for Health" programme). The midwife on call, the driver, and the maternity ward at Brikama were contacted by Traditional Birth Attendants (TBAs) and Village Health Workers (VHWs) using mobile phones (see below) if there was an emergency involving a pregnant woman or girl, a baby or young child (<5 years) or any patient with major trauma. After any necessary stabilisation, the patients were transferred to either Brikama Hospital or RVTH.

To enable TBAs and VHWs to contact the emergency ambulance service, each was equipped with a mobile telephone containing a SIM card donated by one of the mobile phone network providers (Gamcel).

From 14th May 2007 to 1st March 2010, 109 patients were transferred to Brikama or RVTH or were managed at home (Table 1)

**Table 1:** Emergency ambulance data for Brikama region from 14/5/07 until 20/2/10

Patient numbers	Total	Emergency	Resuscitation given	Outcome
1,52,63	3	Obstructed Labour	IV glucose x3, oxygen x1, normal saline bolus x2, CS x3	3 mothers alive, 1 infant death

8,12,13,18,19,22,25,29,30,31,32,34,37,41,46,56,57,68,99,103,	20	Ante-partum Haemorrhage or miscarriage	CS × 6, IV glucose × 2, IV Normal saline × 19, lateral tilt × 1, oxygen × 2, leg elevation × 2, blood transfusion × 1, IV antibiotics x1	All mothers alive, 7 infant deaths

3,40,43,45,85,91,94,101	8	Eclampsia and 2 cases of severe pre-eclampsia	IM/IV hydralazine × 4, IV magnesium sulphate x6, CS x2, Diazepam × 1, oxygen x1, airway opening x2, oropharyngeal airway, suction x2	7 mothers and infants alive, 1outcome unrecorded

1,2,10, 14,36,45,54,93,100,101,104,	11	Pregnancy Induced Hypertension without severe pre-eclampsia or eclampsia	IV hydralazine × 6, IM hydralazine × 2, CS × 3,	11 mothers and 11 infants alive

4,10,15,16,44,53,59,72,76,77,84,89,	12	Twins × 4, breech × 6, or compound presentation × 2	IV glucose × 5, CS x4, vaginal breech x4, IV normal saline × 5, blood transfusion × 1	11 mothers alive, 1 breech fractured femur, 4 fresh stillbirths (one breech head could not be delivered) 1 outcome unrecorded

7,10,16,17,20,26, 27, 28,33,50,66,69,82,86,87,88,90,101,102	19	Prolonged labour	IV glucose × 16, IV antibiotics x1, IV normal saline × 3, ventouse at home × 2, urinary catheter × 1, CS × 9,	19 mothers alive 1 stillbirth

65,81,83,92,96,97,100,106	8	Postpartum haemorrhage	IV normal saline x5, misoprostol x2, ergometrine × 4, oxytocin IV × 1, blood transfusion × 5, IV antibiotics × 2, repair tear × 1	7 mothers alive 1 outcome unrecorded

39, 53,58, 83,97,	5	Severe anaemia	Oxygen × 1, blood transfusion × 5, IV normal saline × 3, IV antibiotics × 1	5 mothers alive

20,21,36,51,55,62,70,71,73,74,75,87,93,102,107	15	Primagravida	IV 50% glucose × 7, IV normal saline × 4, CS × 4, ventouse × 1	15 mothers alive 1 stillborn infant

38,42,47,49,53,66,67,78,79,80,88,90,106,109	14	4 or more previous births	IV normal saline × 4, IV glucose × 3, blood transfusion × 1	13 mothers alive 1 unrecorded 2 stillborn (twins)

26,75,95	3	Infection in pregnancy	IV 50% glucose x1, IV antibiotics × 3, CS x1	3 mothers alive 1 stillborn infant

5,29,45,64,84	5	Preterm labour		5 mothers alive 1 infant stillborn

9(3 patients),11(2 patients),108	6	Road accident	Airway opening x3, compression for bleeding x1, IV normal saline x2, Pain control,	4 survived and 2 died

6(3 years),24(3 weeks),35(8 days), 61(2 years)	4	Paediatric emergency	Airway opening × 2, oropharyngeal airway × 1, IV Normal saline x2 IV glucose followed by infusion × 1, IV antibiotics × 1, Assisted ventilation with bag-valve-mask and oxygen x1, compression to stop bleeding × 1, blood transfusion x1	4 survived

23,39,48,60,64,98,105	7	Miscellaneous (asthma, loss of consciousness, severe vomiting, retained placenta, dehydration and shock, staff member in labour)	Anti-asthma treatment x2(salbutamol, hydrocortisone, aminophylline), oxygen x1, IV normal saline x2, IV glucose x3, retained placenta removed after urinary catheter x1, IV antibiotics × 1	6 pregnant mothers and their infants survived. One man with dehydration and shock died

Abbreviations: IM = intramuscular injection, IV = intravenous, CS = Caesarean section

Initially most patients went to RVTH, but when the operating room and blood bank had been established at Brikama and, when a nurse anaesthetist and surgeon were available, those requiring surgical care went directly to Brikama Hospital.

From December 2008 to January 2010, there was no surgeon in The Gambia able to cover the work at Brikama Hospital and all transfers were made to RVTH. Fortunately, since February 2010, a Cuban surgeon has been operating at Brikama Hospital.

A shortage of charging facilities meant that the batteries in the initial mobile phones used for summoning the emergency ambulance soon lost their power. Telephones with a longer battery life were introduced (Nokia 3310) and one village acquired a solar charging system. At times mobile phones had no credit available and Community Health Nurses, responsible for community sensitisation regarding emergency care and support for TBAs and VHWs, reported that many villages had taken on the responsibility for providing credit for the emergency calls until free-phone numbers can be negotiated with network providers to help sustainability of this communication system (discussions currently underway).

### 3. Training in the recognition and management of emergencies

Doctors, nurses, midwives and medical students, providing emergency care 24 hours a day on the "front line" in the hospitals and health centres throughout the Gambia, were trained using an ALSG accredited educational approach involving lectures, skill stations, workshops and scenarios. The aim was to improve the management of an emergency as soon as possible after its presentation to achieve the best outcome for the patient, as well as to identify as early as possible an **impending **emergency which if not recognised and managed rapidly and appropriately leads to collapse and possible death, permanent brain or other major organ injury.

The curricula and training materials for the training courses were adapted to meet local needs from those developed in earlier work in Pakistan [13] and from emergency maternal and child life support courses widely taught in well-resourced countries [12]. The courses were integrated with teaching materials of WHO, such as the IMEESC toolkit, and publications from the "Making Pregnancy Safer" [18,19] and "Child and Adolescent Health" [20,21] departments. The resulting teaching materials for these structured trainings are accredited by ALSG and are available for download on the MCAI website [22]. Specialised training equipment such as manikins, computers, multimedia and overhead projectors, were provided.

3-6 weeks prior to each training course, each candidate received the training manual [22].

Pocket books containing the essential components of emergency care were also provided for all health workers managing emergencies [22]. Both manuals and pocket books were translated into Spanish [22] to assist the Cuban doctors who constitute almost all doctors working in the public health service in The Gambia.

Training courses for "providers" of emergency care were initiated by volunteer UK based senior instructors from ALSG. Initially there was a programme lasting one working week encompassing emergencies in all age groups and including major trauma. Health professionals attending this course were chosen by the MOH to represent some of the most senior and able in the country. Almost all were midwives and registered nurses with a small number of doctors. Because of the scarcity of trained staff and therefore difficulties in providing cover for the hospitals over a full working week, the original course was subsequently divided into 2; each of 3 days. The first course covers emergencies in pregnant mothers and newborns (Emergency Maternal and Neonatal Healthcare -EMNH) and the second course covers emergencies in infants and children (Emergency Child and Trauma Healthcare-ECTH), the latter placing a particular emphasis on major trauma management. The aim was to ensure that as many staff as possible in The Gambia, initially all of those based in the Brikama region, undertook both courses,

During the initial "provider" courses, the volunteer UK ALSG Instructors selected successful trainees from each course who had high achievement and potential skills in teaching to undertake the Generic Instructors Course (GIC) and if successful become accredited ALSG Instructors. The GIC is an educationally approved [23] two day course to 'train trainers' and is widely taught in well-resourced countries. Once these Trainee Instructors had successfully completed the two day GIC course and completed their instructor candidacy (monitored by UK instructors), a pool of accredited 'in-country' Gambian ALSG Instructors was created, essential for the programme's sustainability, in-country ownership and country-wide coverage.

By March 2010, 217 midwives, nurses and doctors had completed "provider" courses in emergency care. 53 individuals were identified as potential instructors of which 33 have undertaken Generic instructor Courses and 15 completed apprenticeship to become fully accredited ALSG instructors.

Two senior midwives (FF and Mr S Jammeh) have become The Gambia's first course directors.

Recently a proportion of the Cuban and newly qualified Gambian doctors have undergone EMNH courses and are soon to receive ECTH training.

The training programme for TBAs, who are present at most home births, focused on their early recognition of maternal and neonatal problems and the development of a system which as rapidly as possible gave access to skilled midwifery support through the emergency ambulance system described above.

**Figure 1:**
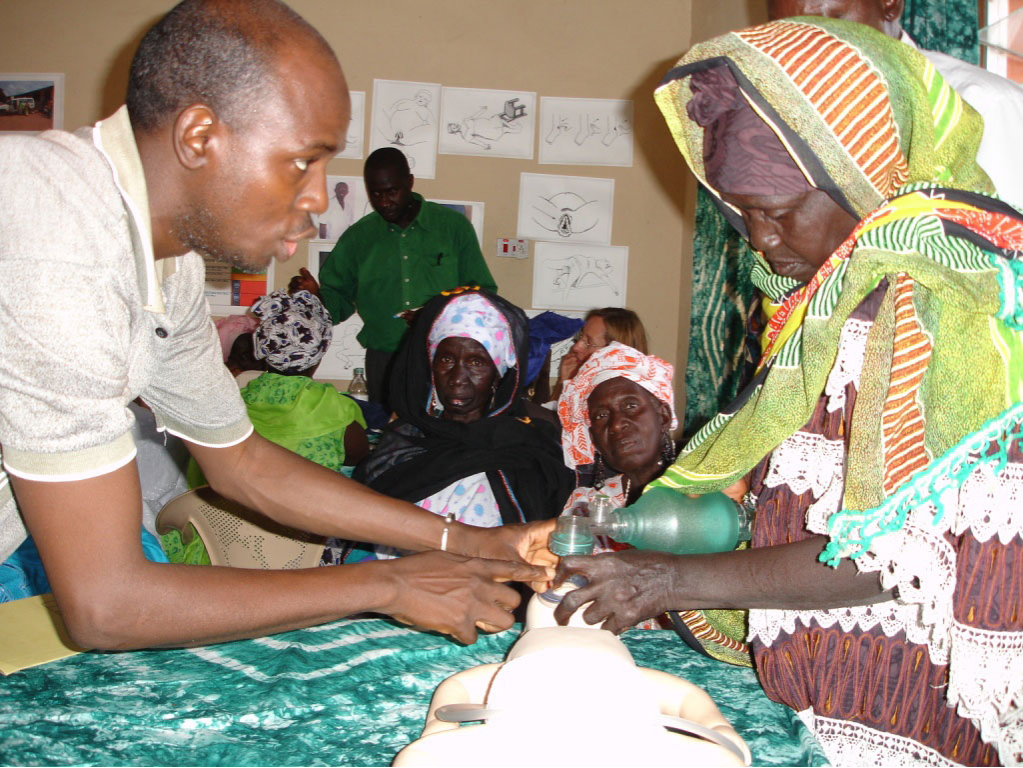
**Training of a Traditional Birth Attendant in bag-valve-mask ventilation of the newborn**. A Gambian instructor is helping a Traditional Birth Attendant (TBA) to undertake bag-valve-mask ventilation on an infant manikin. The TBA will need to breathe for a newborn infant requiring resuscitation after first drying the baby and opening his/her airway.

Evidence shows that wherever maternal mortality was significantly reduced, both in developed and disadvantaged countries, the majority of deliveries are attended by skilled personnel [24] and this is the ultimate aim of WHO. Unfortunately, it has been estimated that it will take many years and unspecified funding to train sufficient skilled birth attendants for this to occur. In the meantime, women and babies continue to die. TBAs are accessible and culturally well accepted, but do not have life-saving skills. WHO have recommended the following: *"Where TBA training is undertaken, it should be part of a broader strategy that includes a built-in mechanism for referral, supervision, and evaluation*" [25]. The availability of TBAs trained to recognise and initially resuscitate patients with emergencies was particularly important given the situation in the Gambia where so many babies are born at home and where the TBAs undertake about half of all deliveries in the country. Resuscitation of those newborn infants who do not breathe at birth must be undertaken immediately if it is to be successful. The TBAs are present when and if a baby does not breathe and therefore they were equipped with bag valve mask resuscitators and trained with evidence from their actions with high quality manikins that they could appropriately perform resuscitation at birth. (Figure 1)

VHWs are volunteer workers of both sexes (TBAs are all women). They receive a basic training from the government, largely in managing nutrition and preventing disease. Discussions showed they felt inadequately prepared when called to attend emergencies, especially convulsions and trauma. The VHWs were therefore taught basic life-saving techniques..

The courses for TBAs and VHWs, supplemented by graphic manuals [22], focused on recognition of and immediate response to emergencies that could not wait for the arrival of skilled support. Courses were undertaken in local languages by the Gambian faculty of instructors supported by UK ALSG instructors (BP, DW, MB, BH and DS).

At the end of each course, each TBA and VHW successfully completing the course were issued with a bag valve mask for the resuscitation of the newborn infant and a digital thermometer and chart to aid recognition of puerperal or neonatal sepsis. VHWs were additionally provided with a pocket-mask, cling-film for burns, triangle sling for upper arm fractures (and shown how to make replacements), crepe bandage for snake bites, and taught how to make various sizes of wooden splints (to include joint above and below fracture) for lower limb fractures. They were given wadges of gauze for haemorrhage control (and suggested to replace these with clean rags), gloves and mobile telephone.

83 TBAs and 48 VHWs were trained.

WHO Gambia provided fellowships for the training of 10 nurse anaesthetists and 11 peri-operative nurses. The training was coordinated by the Head Nurse Anaesthetist at RVTH (MB). The training was extended to include introduction of spinal and other regional anaesthesia by a volunteer consultant anaesthetist from MCAI and ALSG (DW).

### 4. Monitoring and evaluation

Training was continuously evaluated by the in-country team, the International ALSG volunteers and the 'in-country' Programme Directors (RC-C, MC, FF and Mr S. Jammeh) using course evaluation forms, attendance, assessment and registration records, training reports and logbook analysis (see below).

Each doctor, nurse or midwife completing EMNH and ECTH "provider" courses received a logbook for recording details of each subsequent emergency they managed. "Emergency" was closely defined as conditions of Airway, Breathing, Circulation or Conscious level that would likely lead to death or severe brain damage in 6 hours if not given immediate and appropriate emergency treatment [13].

From February 2007 (when they were first issued) until July 2009 monitoring of the resuscitation log books was undertaken. Since staff moved around the country as part of their work in the public health service, this monitoring was country-wide. 293 resuscitations were recorded. (Table 2)

**Table 2:** Details of resuscitations documented in logbooks by providers of emergency care

CONDITIONS RESUSCITATED	TOTAL	DEATHS
Ante partum haemorrhage	17	

Postpartum haemorrhage	**20**	

Massive haemorrhage	**2**	

Eclampsia	**23**	

Severe anaemia	**21**	

Shock	**22**	**3**

Severe sepsis including malaria	**33**	

Road accident	**7**	

Severe pneumonia	**10**	

Birth asphyxia	**45**	**3**

Burns	**9**	

Complications of labour/delivery	**12**	**2**

Major medical problems (epilepsy, diabetes, airway obstruction, severe dehydration, severe anaemia, malnutrition, apnoea, hypoglycaemia, heart failure, hypertension, liver disease, gas poisoning)	**17**	**3**

Major trauma (gunshot, stabbing, fracture, head injury)	**9**	**1**

Severe asthma	**10**	

Unknown	**36**	**1**

**TOTAL**	**293**	**13**

A log of each patient attended by the emergency ambulance was also kept.

Critical incident mortality/morbidity meetings were introduced for reviewing deaths and "near misses" to identify and put in place system change or re-training to support continued quality improvement.

The first meeting discussed a case of ruptured uterus and resulted in a number of country-wide recommendations to decrease the risk of a recurrence. In another patient with a post-partum haemorrhage, the weekly quota of fuel available for the emergency ambulance had been used up leading to a major delay in its departure and in the provision of emergency care. Following such a meeting, attended by most of the staff, including the driver, it was agreed that a contingency fund would be established to prevent a recurrence of this scenario.

The following were some of the key recommendations from these meetings:

• Need for oxygen in ambulances (currently being implemented country-wide)

• Great care and close observations when oxytocin is used in multiparous mothers in labour

• More blood available in the blood bank

• Calcium gluconate to be always available as an antidote for magnesium sulphate toxicity

• Misoprostol to be available in all health facilities

• More training in the use of intra-uterine tamponade catheter for post partum haemorrhage

Meetings were held on the emergency ward so that patient care was not impaired by an absence of staff.

An interim evaluation of the programme was undertaken by Dr A MacFarlane in May 2008 [26].

### Sustainability

All efforts are being made to develop a sustainable project, however:

1. It was not possible to find a surgeon/obstetrician to work at Brikama Hospital for a period of 12 months, despite a well-equipped operating theatre, nurse anaesthetist and peri-operative nurses.

2. Lack of trained healthcare staff is one of the greatest challenges faced by this programme. A lack of doctors is being partially addressed by the recently established medical school. This lack of staff and inadequate funding of the public sector means all existing health workers are faced with enormous workloads leading to burn out, low morale and attrition to the private health sector or to non medical occupations which pay better and do not require such hard work and frustrations. In addition international organisations employ Gambian trained professionals, which reduces the number of nurses and midwives available to the public service even further [27]. Steps to address these problems are underway.

3. The living conditions for health workers in the hospital are atrocious and, in retrospect, it was inappropriate to renovate the hospital without, in parallel, paying similar attention to staff accommodation.

4. A high workload and lack of morale amongst nurses also leads to poor attention to protocols, little time for patient care, and almost non-existent documentation of clinical signs or hospital notes.

5. The high mortality rates also add to poor morale. More attention needs to be paid to support for the staff working in the NHS.

6. A lack of funds for fuel has implications for emergency healthcare which can only be helped by the availability of more central government funding for the healthcare sector.

### Additional developments

• a new one day course of training for senior nurses and doctors in the use of morphine for treating severe pain. Morphine is rarely used despite its low cost. Reasons for this include the logistic efforts required to control and monitor its use and the possibility of misuse.

• a one day course on the recognition of the clinical signs of child physical and sexual abuse

• a one day course for ward cleaners on infection control, including an enhancement of their duties to include the training of patients/relatives in hygiene.

• attempts to establish blood banks with national programmes all over the country to decrease avoidable deaths due to haemorrhage and severe anaemia. One of the main problems has been the high and prohibitive costs of suitable refrigerators and frequent loss of mains electricity, resulting in variable periods without power. The use of a generator to maintain power in the hospitals 24 hours a day has proved too expensive in terms of fuel and a new technique based on a small pharmacy refrigerator designed for use in tropical countries that requires only 110 watts of power, runs from a large battery (220 ampere hours) with an inverter and automatic switching system and supported by solar power is under development.

### Conclusions

This partnership between the Gambian MOH, WHO and two international medical organisations to strengthen emergency healthcare systems has combined medical education with improved resources and health service development, thereby enabling the trained healthcare workers to use their newly acquired skills and knowledge, rather than continue the frustration caused by the absence of life-saving drugs and equipment. The programme has also integrated emergency care from the community through to the first referral and tertiary hospitals strengthening the "emergency chain of care" [28]. By working as partners, and including the Ministry of Health as the lead, the aim was for the programme to become an integral part of The Gambian public health system on which 95% of the population rely. Every effort has been, and will continue to be, made to develop sustainability as the project model is spread through the country. This will be achieved with advice from the independent evaluation, through further partnerships with local, national and international agencies. This is a long-term project with no rapid solutions.

Our hope is that this system template can be replicated in other poorly resourced countries.

## Competing interests

DS, BP, BH and DW received reimbursement of expenses for travel and accommodation from MCAI and ALSG during their visits to The Gambia. All other authors declare that they have no competing interests.

## Authors' contributions

RC, MC, NS, MB were responsible for initiating the programme in The Gambia. DW, FF, MC, MD, SW, BP, BH and DS have produced the education materials and coordinated the training programmes. MB and DW have setup the anaesthetic components. BP, RC, MC, NS and DS coordinated the programme with the close assistance of AS. RM was one of the main writers of this paper. All have contributed to drafting the paper and ensuring the data have been collected and presented as accurately as possible. All authors have read and approved the final manuscript^.^
